# COVID-19 Vaccine Roll-Out in South Africa and Zimbabwe: Urgent Need to Address Community Preparedness, Fears and Hesitancy

**DOI:** 10.3390/vaccines9030250

**Published:** 2021-03-12

**Authors:** Tafadzwa Dzinamarira, Brian Nachipo, Bright Phiri, Godfrey Musuka

**Affiliations:** 1 Department of Public Health Medicine, University of KwaZulu Natal, Durban 4001, South Africa; 2 ICAP @ Columbia University, Pretoria 0157, South Africa; bp2483@cumc.columbia.edu; 3 AIDS and TB Unit, Ministry of Health and Child Care, Harare, Zimbabwe; bnachipo@gmail.com; 4 ICAP @ Columbia University, Harare, Zimbabwe; gm2660@cumc.columbia.edu

**Keywords:** COVID-19, vaccine, hesitancy, Africa

## Abstract

South Africa became one of the first African countries to receive the COVID-19 vaccine. As the rest of Africa prepares to receive COVID-19 vaccines, most countries in Africa have set up national-level coordination committees for developing national vaccination deployment plans. While the main focus of these committees has been on setting up strategies that facilitate the swift distribution of COVID-19 vaccines once they are available, the role of effective public health awareness should not be ignored. Countries must devise strategies on how best to enhance public understanding and curb misinformation about the vaccines. With this viewpoint, we unpack the threat of COVID-19 vaccine hesitancy and offer recommendations for COVID-19 vaccine communication strategies in the South African and Zimbabwean contexts.

## 1. Introduction

In December 2019, a pneumonia-like illness of unknown origin was detected in Wuhan, China. The causative agent was identified and named severe respiratory coronavirus 2 (SARS-CoV 2) by early February 2020 [1]. Transmission routes for SARS-CoV-2 have been grouped as contact [2], droplet [3], airborne [4], fomite [5], fecal–oral [6], bloodborne [7], and animal-to-human transmission [8]. Infection with SARS-CoV-2 primarily causes respiratory illness ranging from mild disease to severe disease and death, while some people infected with the virus never develop symptoms. The most common symptoms of COVID-19 are fever, dry cough and tiredness [9]. As of 20th February 2021, there have been more than 110 million confirmed infections and over 2.46 million confirmed deaths globally. By the same date, although with concerns of under reporting [10,11], Africa has reported over 3.8 million confirmed cases with over 100,000 deaths [12]. South Africa has reported over 1.5 million cases with over 49,000 deaths [12]. Zimbabwe has reported over 35,000 confirmed cases and over 1400 deaths [12].

In Sub-Saharan Africa, COVID-19 has resulted in additional pressure to already strained health systems characterized by poor health outcomes with high mortality rates linked to a triple burden of disease (HIV, tuberculosis, and non-communicable diseases) [13]. In an effort to curb COVID-19 transmission, most African countries imposed movement restriction such as quarantine (lockdown) [14]. The lockdowns came with unintended consequences, such as widening economic inequalities, mental health problems, and exacerbating poor medical outcomes that are not COVID-19 related [13,15,16]. The overwhelmed health systems, the disruption of other health services [17,18], and the economic impact of lockdowns in the region [19], have presented complications that have resulted in varying COVID-19 responses in the region. The availability of COVID-19 vaccines has presented countries with a unique opportunity in the COVID-19 response. In addition to the primary effect of reducing disease burden, widespread vaccination will allow countries to lift restrictions previously imposed to control the spread of the virus and revive ailing economies, whilst enabling people to regain their “normal” lives.

Vaccination has been reported as one of the top notable public health achievements to have occurred during the 1900s. Vaccination has resulted in the eradication of smallpox and control of poliomyelitis, measles, rubella, tetanus, diphtheria, and other infectious diseases [20]. Despite all the public health successes in reducing the spread of infectious diseases through vaccines, a large portion of the global population still expresses concerns about the safety, efficacy, and need for vaccines, a phenomenon known as vaccine hesitancy [21]. With this viewpoint, we unpack the threat of COVID-19 vaccine hesitancy, and offer recommendations for COVID-19 vaccine communication strategies in the South African and Zimbabwean contexts.

## 2. COVID-19 Vaccine Availability in South Africa and Zimbabwe

There has been global competition to procure COVID-19 vaccines, and African countries have been substantially less successful than richer countries in securing supplies. According to the Secretary General of the United Nations, 75% of all COVID-19 vaccines have been administered in just 10 countries [22]. According to the Duke Global Health Institute, as of mid-January 2021, high income countries, which represent only 16% of the world’s population, have already purchased over 60% of the COVID-19 vaccine doses [23]. For instance, Canada has purchased enough doses to vaccinate its entire population five times; the European Union and the United States of America have already ordered enough to vaccinate their entire populations 2.7 and 2 times, respectively [23]. In the same time period, the African Union had only ordered enough doses for 38% of its population [23]. At present, African countries which have received COVID-19 vaccines have largely done so through direct purchases from manufacturers, or as donations from countries such as China, Russia, India and the United Arab Emirates (UAE). In Sub-Saharan Africa, only five countries have rolled out COVID-19 vaccination programs, namely Mauritius, South Africa, Seychelles, Rwanda and Zimbabwe [24], with the limited vaccine dose supplies being prioritized for high-risk groups, including frontline workers.

Most African countries are expecting to obtain the COVID-19 vaccine through the COVAX facility. COVAX was launched in April 2020 by the World Health Organization, the European Commission, and France as a global response strategy to the COVID-19 pandemic [25]. COVAX was established as a global initiative to maximize chances of successfully developing COVID-19 vaccines and manufacturing them in the quantities needed to end this crisis, and in doing so ensure that ability to pay does not become a barrier to accessing them. The COVAX facility aims to ensure both equitable and swift access to the COVID-19 vaccine in 190 countries across the globe. In particular, COVAX aims to deliver at least 2 billion doses by the end of the year, including at least 1.3 billion doses to 92 lower income economies [26]. However, in spite of their equitable goals, no deliveries have been made yet in Africa at the time of writing.

### 2.1. South Africa

On 1 February 2021, South Africa became one of the first African countries to receive a COVID-19 vaccine. The country received a million doses of the AstraZeneca/Oxford COVID-19 vaccine, produced by AstraZeneca-SK Bioscience (AZ-SKBio) and the Serum Institute of India (AZ-SII) [27]. The roll-out of the AstraZeneca/Oxford COVID-19 vaccine was suspended on the 8th of February 2021 following the release of results that showed the vaccine has low efficacy against the 501Y.V2 variant of this coronavirus, the variant most common in the South African population [28]. On the 17th of February 2021, South African began a roll-out of the Johnson and Johnson COVID-19 vaccine with an initial 80,000 doses [29]. In addition to being effective against the 501Y.V2 variant, this vaccine, compared to AstraZeneca/Oxford and other currently available ones, is cheaper and requires only regular refrigeration for storage. The country has secured a total of 9 million doses of the Johnson and Johnson COVID-19 vaccine with 20 million doses of the Pfizer/BioNTech expected at the end of the first quarter of this year.

### 2.2. Zimbabwe

Zimbabwe received its first delivery of a COVID-19 vaccine on the 15th of February 2021 with the roll-out of the vaccination program beginning 18th February 2021 [30]. At the time of writing, the BBIBP-CorV vaccine, produced by the Beijing Institute of Biological Products and Sinopharm, is approved for use in Bahrain, China, Egypt, and UAE. Zimbabwe received 200,000 doses as a donation from the Chinese government with a further 600,000 doses bought by the Zimbabwean government expected to arrive in March 2021. The doses received have been prioritized for frontline workers, especially medical personnel, the elderly, and those with underlying conditions. Zimbabwe expects to receive a donation of 75,000 doses of the COVID-19 vaccine from the Indian government. The country aims to inoculate at least 10 million of its 16 million citizens to achieve herd immunity. As of 2 March 2021, two weeks into the vaccination program roll-out, only 25,000 doses had been administered to healthcare and other frontline workers [31].

## 3. The Threat of COVID-19 Vaccine Hesitancy in South Africa and Zimbabwe

Herd immunity, which offers some protection to unvaccinated individuals, is compromised when widespread vaccine acceptance is not achieved, and disease outbreaks result [32]. More than 39 million South Africans (67.25% of the population) need to be vaccinated to achieve herd immunity [32,33]. However, a recent survey by financial technology company CompariSure reported 52% of South Africans will not take the COVID-19 vaccines with religion, fear of needles, and unconsented government tracking being reported as some of the deterrents [34]. Similarly, a Zimbabwean COVID-19 vaccine hesitancy survey preliminary report revealed that 50% of Zimbabweans would accept the vaccine while 30% and 20% were unsure and would reject, respectively [35].

Several communities in Africa have always resisted vaccines, irrespective of the type and form of vaccination. For instance, in Zimbabwe, the Apostolic Faith community religious group, which makes up a third of the population, is historically known to have poor health-seeking behavior, including vaccine uptake [36,37,38]. Recent studies on health-seeking behavior of the apostolic groups in Zimbabwe suggest attribution of the causation of disease to spiritual factors negatively shapes healthcare-seeking behavior [36,39]. There is an urgent need for the government to effectively overcome theological rigidity on health-related issues among the apostolic sector as it negatively affects any vaccination program. Targeted information, education and communication materials, and promotional events can address misinformation, myths, and lack of understanding on vaccination in general and more specifically for COVID-19. In South Africa, a mega-church preacher recently spoke publicly rallying his followers to not accept the COVID-19 vaccine [40]. The influence of such individuals in society should not be ignored. In northern Nigeria, vaccine hesitancy due to low-risk perception and religiously motivated myths have been a constant threat to polio eradication [41].

The halt in South Africa’s roll out of the AstraZeneca/Oxford vaccine described above [28] may further diminish public trust in COVID-19 vaccinations, as an impression is being generated that vaccines may not be effective after all—specifically for 501Y.V2 but also in general. In Zimbabwe, the 501Y.V2 variant now accounts for more than 60% of COVD-19 cases [42]. The roll-out of the Sinopharm vaccine in Zimbabwe may face poor acceptance due to the lack of publicly available evidence on its effectiveness against the 501Y.V2 variant.

The sources of health- and vaccination-related information play vital roles in the choices people make about vaccinations, with current research pointing to information overload [43], misinformation, and myths on the internet and social media platforms [44] as potential threats to vaccine uptake. COVID-19 is the first pandemic in history in which technology and social media are being used on a massive scale to keep people safe, informed, productive, and connected. At the same time, the technology we rely on to keep connected and informed is enabling and amplifying an infodemic that continues to undermine the global response and jeopardizes measures to control the pandemic [45].

The ongoing global discussion on treatment options for COVID-19 may potentially leave communities undecided on whether to trust the treatment option versus the vaccination option. In Zimbabwe, debate among scientists and researchers has been raging recently about the efficacy of ivermectin in the treatment of COVID-19. Some medical doctors have been widely advocating for its use. This discussion has the potential to swerve communities towards the treatment options versus vaccines. In South Africa, some health workers have started to question the efficacy of the COVID-19 vaccine [46]. Similarly, in Zimbabwe, nurses have been reluctant to get vaccinated [47]. Frontline workers are a major conduit of correct information. When there is a lack of clarity, uncoordinated approaches, and differences amongst the public health experts, COVID-19 vaccine hesitancy among communities will be unavoidable. A similar impact is seen when COVID-19 vaccine misinformation originates from individuals holding high-ranking positions in government [48].

As noted by polio vaccination hesitancy research in northern Nigerian states, failure by the governments to deliver on key areas of their mandate such as security, water, sanitation, and food security may breed mistrust in other areas such as vaccines [49,50]. In these contexts, communities are likely to perceive any interventions provided by the government with suspicion. For the COVID-19 vaccine, these beliefs include that it was most likely procured without following due process or that the available vaccine has poor efficacy as communities no longer believe that the government is able to make decisions in their best interest. Zimbabwe may fit into this model as decades of poor service delivery [51] and cases of high-profile corruption within the COVID-19 national response [11] may compromise communities’ trust in the vaccine.

The threat of vaccine hesitancy is not restricted to our study context alone [52]. In France, a sample of working-age adults expressed vaccine hesitancy for vaccines manufactured outside the European Union [53]. The same study revealed that a lower perceived severity of COVID-19, gender, age, lower educational level, poor compliance with recommended vaccinations in the past, and no report of specified chronic conditions as factors associated with outright vaccine refusal [53]. Low COVID-19 risk perception was also reported as an important factor associated with vaccine hesitancy in Italy [54]. A study conducted in Ireland and the United Kingdom found that vaccine hesitancy/resistance was evident for 35% and 31% of these populations, respectively [55]. The same study revealed that for both populations, those resistant to a COVID-19 vaccine were less likely to obtain information about the pandemic from traditional and authoritative sources and had similar levels of mistrust in these sources compared to vaccine-accepting respondents [55]. These findings from other countries in different geographical locations and socio-economic characteristics underscore the need to prioritize the threat of COVID-19 vaccine hesitancy in the African context.

## 4. Steps to Ensure COVID-19 Vaccine Acceptance in South Africa and Zimbabwe

### 4.1. Journey Mapping

**Figure 1:**
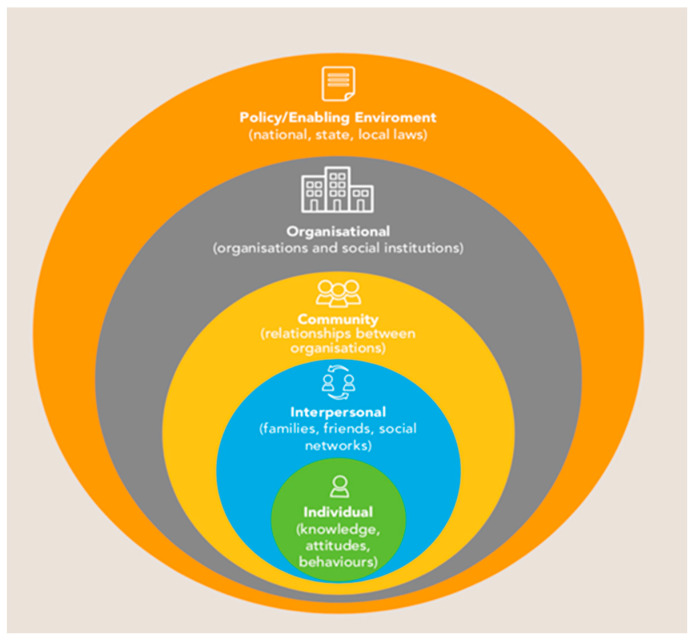
Socio-ecological model [57].

Mapping the journey that each individual or communities goes through in their quest to seek good health is important in improving community preparedness and addressing fears towards the COVID-19 vaccine. By looking at the lives of specific target groups and following their health-seeking behavior, policy makers will be able to tailor messaging to meet the needs of each target audience [56]. Continuous mobilization and community engagement using simple non-medical terms at an individual level will go a long way in ensuring COVID-19 vaccine acceptance. In this regard, the socio-ecological model (Figure 1) plays a critical role in shaping social norms and beliefs in the two countries’ contexts from the individual level (knowledge, attitudes, behaviors) to the interpersonal level (family, friends, social networks), through to the community and beyond. The general preparedness of communities to receive and accept the vaccines needs to critically take this model into context. Direct application of this model to the development of communication interventions for COVID-19 vaccine acceptance will ensure mobilization and commitment of political and social resources for change at the political, social, and individual levels.

### 4.2. Addressing Risk Perception as a Priority

The low effect at a personal level of COVID-19 in its first wave in Africa contributed to a widespread belief that COVID-19 is overwhelmingly not an African problem [58]. However, the acceptance of the COVID-19 vaccine itself is premised on the risk perception that communities have of COVID-19 as a threat to their health, families, and livelihoods. In Zimbabwe, Population Services International (PSI) launched the “COVID is closer than you think. Take Care!” campaign to educate communities on the risk of COVID-19 infection [59]. Intensified campaigns to increase the risk perception of COVID-19 infection will pave the way and heighten the need for and acceptance of vaccines.

### 4.3. Positioning the COVID-19 Vaccine as a Tool for Economic Strengthening

A strong healthcare brand position communicates the brand’s value position with consistency, clarity, conviction, and credibility in a way that has meaning for consumers and is unmatched by others [60]. Clear positioning of the vaccine in the context of the primary needs of the target audience will help in swaying the perception of the value of the COVID-19 vaccines. The benefits of the vaccine should be communicated from the perspective of the individual and what is of high value to them (family, health, welfare, lifestyle).

The second wave of COVID-19 has seen Zimbabwe go through another intense total lockdown, with a ban on all informal trade, in a country where a large proportion of the population largely lives from “hand to mouth”. In this instance, the COVID-19 vaccine should be positioned as an opportunity that can enable small to medium traders to get their lives back and once again trade as normal. Correctly positioning the emotional and immediate economic benefits of the COVID-19 vaccine to the individual will likely sway their acceptance of the vaccine.

An analogy can be made withthe Voluntary Medical Male Circumcision program in Zimbabwe where initially the intervention was positioned as a medical program that would help reduce the chances of men contracting HIV by 60% [61]. Despite this being factual, it however led to some level of stigma and apathy in acceptance of circumcision in Zimbabwe amongst adolescent and adult males. Awareness of this benefit stood at 90%, but the willingness to get circumcised was at 50% [61]. However, re-positioning the intervention by changing the key benefit presented to improving the hygiene of men resulted in improved uptake. The same lessons could be learned and applied to the COVID-19 vaccine and the benefits positioned in the context of the immediate needs and values of the target populations.

### 4.4. Segmentation of the COVID-19 Vaccine Market

Conducting social “market” research will allow the unearthing of critical issues such as underlying barriers and facilitators for COVID-19 vaccine compliance in the target group. Social “market” research goes beyond the traditional demographic methods of segmenting audiences, and digs deeper into the psychographic issues, enabling comprehensive segmenting of the audience members and instituting appropriate and targeted messaging and interventions for each segment. This research into COVID-19 vaccine attitudes in the two countries can help design communications and enhance understanding of the target audience’s characteristics, attitudes, beliefs, values, behaviors, and determinants, benefits, and barriers to behavior.

### 4.5. A Coordinated and Strategic Approach to COVID-19 Vaccine Communication

Most countries in Africa have set up national-level coordination committees for developing national vaccination deployment plans. While the main focus of these committees has been on setting up strategies that facilitate the swift distribution of COVID-19 vaccines once they are available, the role of public health awareness should not be ignored.

For COVID-19 vaccine communication strategies, firstly, we recommend joint efforts between government agencies and civil society to reach the communities. Civil society Organizations (CSOs) have historically been pivotal in mobilizing the support of the public through working directly with vulnerable populations and the invaluable access and reach they have, particularly to some of the hard-to-reach communities, whether they are in remote rural villages or urban settings. Further, CSOs will play an important role because of their ability to establish trust at the grassroots level, with communities, families, and individuals [62]. When engaged, CSOs have the capacity to complement government efforts to ensure the preparation of local communities’ awareness and ultimately acceptance of the COVID-19 vaccine.

Secondly, governments should utilize popular online social media platforms, such as WhatsApp and Twitter, to raise public awareness of the benefits of the COVID-19 vaccine. Whilst social media is dominant in certain population groups, it is imperative to note that different audiences have varied preferences for receiving credible information about personal health. Therefore, the development of public health campaign materials specifically tailored to social media platforms and their users while increasing the use of emotive language and imagery that is common to social media may also help raise COVID-19 vaccine awareness [63]. To further improve the efficacy of social media campaigns, regulations and policies should be set up to address unscientific false claims on the COVID-19 vaccines that may be spread by anyone, be it politicians, health professionals, scientists, or academics [64].

Thirdly, traditional media platforms will play a key role in public approval of the COVID-19 vaccinations. Traditional media includes television, radio, newspapers, magazines, medical journals, books, and pamphlets. This type of media and mass communication was available before the advent of digital media and still has substantial value in reaching the population in both South Africa and Zimbabwe. The impact of how the media constructs and frames messages about vaccination programs has been well elaborated elsewhere [65]. We call on the governments to strengthen utilization of traditional media to target those within the population that may not have access to or interest in digital media.

Fourthly, the engagement of popular, influential individuals in society, such as musicians and/or the clergy, should also be explored [66,67,68]. Zimbabwe and South Africa are characterized by mega-church preachers who draw large number of followers and as described above their influences can be counter to vaccine acceptance. Efforts should be made to educate these church leaders so as to ensure they spread the correct information about the COVID-19 vaccines to their followers.

Finally, building on already existing channels, communication strategies should engage representatives of community groups affected by COVID-19. In this way, all stakeholders would have clear guidelines to communicate scientifically sound messages to the public, communicated in simplified language easily understood by the communities. The engagement of representatives of community groups would also allow for feedback mechanisms for the acknowledgement of community efforts in previous health interventions [69] to encourage the acceptance of the prospective COVID-19 vaccine.

## 5. Conclusions

For COVID-19 vaccination in South Africa and Zimbabwe to be successful, approximately 65–80% of each country’s population has to be vaccinated to achieve herd immunity. The threat of vaccine hesitancy cannot be ignored. The factors contributing to vaccine hesitancy in the two countries are multifaceted and thus require equally complex strategies to be addressed. We note that community vaccine hesitancy should be understood and respected. The strategies to overcome it must be guided by the provision of sufficient information using the correct message delivery approaches to the community to allay any concerns they may have regarding the vaccines. The public needs to be aware of all issues concerning COVID-19 vaccines to have the confidence that they are safe. COVID-19 vaccine roll-out programs must be done in a manner that encourages discussions with and engagement of all stakeholders to address and ensure that communities get the correct information to make the correct decision. The national COVID-19 vaccination programs of both South Africa and Zimbabwe could benefit from champions such as artists, politicians, and religious leaders providing the correct information to raise community awareness and ensure vaccine acceptance.

## 6. Disclaimer

The views expressed in this piece are of the authors and do not reflect the official positions of their institutions.

## Data Availability

Not applicable.

## References

[1] WHO Novel Coronavirus (COVID-19). https://www.who.int/emergencies/diseases/novel-coronavirus-2019.

[2] Liu J., Liao X., Qian S., Yuan J., Wang F., Liu Y., Wang Z., Wang F.-S., Liu L., Zhang Z. (2020). Community transmission of severe acute respiratory syndrome coronavirus 2, Shenzhen, China, 2020. Emerg. Infect. Dis..

[3] Zhou F., Yu T., Du R., Fan G., Liu Y., Liu Z., Xiang J., Wang Y., Song B., Gu X. (2020). Clinical course and risk factors for mortality of adult inpatients with COVID-19 in Wuhan, China: A retrospective cohort study. Lancet.

[4] Stadnytskyi V., Bax C.E., Bax A., Anfinrud P. (2020). The airborne lifetime of small speech droplets and their potential importance in SARS-CoV-2 transmission. Proc. Natl. Acad. Sci. USA.

[5] Van Doremalen N., Bushmaker T., Morris D.H., Holbrook M.G., Gamble A., Williamson B.N., Tamin A., Harcourt J.L., Thornburg N.J., Gerber S.I. (2020). Aerosol and surface stability of SARS-CoV-2 as compared with SARS-CoV-1. N. Engl. J. Med..

[6] Xiao F., Sun J., Xu Y., Li F., Huang X., Li H., Zhao J., Huang J., Zhao J. (2020). Infectious SARS-CoV-2 in feces of patient with severe COVID-19. Emerg. Infect. Dis..

[7] Wang W., Xu Y., Gao R., Lu R., Han K., Wu G., Tan W. (2020). Detection of SARS-CoV-2 in different types of clinical specimens. JAMA.

[8] Newman A., Smith D., Ghai R.R., Wallace R.M., Torchetti M.K., Loiacono C., Murrell L.S., Carpenter A., Moroff S., Rooney J.A. (2020). First reported cases of SARS-CoV-2 infection in companion animals—New York, March–April 2020. Morb. Mortal. Wkly. Rep..

[9] CDC Coronavirus (COVID-19). https://www.cdc.gov/coronavirus/2019-ncov/index.html.

[10] Chitungo I., Dzobo M., Hlongwa M., Dzinamarira T. (2020). COVID-19: Unpacking the low number of cases in Africa. Public Health Pract..

[11] Dzinamarira T., Mukwenha S., Eghtessadi R., Cuadros D.F., Mhlanga G., Musuka G. (2020). Coronavirus Disease 2019 (COVID-19) Response in Zimbabwe: A Call for Urgent Scale-up of Testing to meet National Capacity. Clin. Infect. Dis..

[12] WHO COVID-19 in the WHO African Region. https://who.maps.arcgis.com/apps/opsdashboard/index.html#/0c9b3a8b68d0437a8cf28581e9c063a9.

[13] Mhango M., Chitungo I., Dzinamarira T. (2020). COVID-19 Lockdowns: Impact on Facility-Based HIV Testing and the Case for the Scaling Up of Home-Based Testing Services in Sub-Saharan Africa. AIDS Behav..

[14] Dzobo M., Chitungo I., Dzinamarira T. (2020). COVID-19: A perspective for lifting lockdown in Zimbabwe. Pan Afr. Med. J..

[15] Pierre G., Uwineza A., Dzinamarira T. (2020). Attendance to HIV Antiretroviral Collection Clinic Appointments During COVID-19 Lockdown. A Single Center Study in Kigali, Rwanda. AIDS Behav..

[16] Magamela M.R., Dzinamarira T., Hlongwa M. (2021). COVID-19 consequences on mental health: An African perspective. S. Afr. J. Psychiatry.

[17] Nhari L.G., Dzobo M., Chitungo I., Denhere K., Musuka G., Dzinamarira T. (2020). Implementing effective TB prevention and treatment programmes in the COVID-19 era in Zimbabwe. A call for innovative differentiated service delivery models. Public Health Pract..

[18] Mukwenha S., Dzinamarira T., Mugurungi O., Musuka G. (2020). Maintaining robust HIV and TB services in the COVID-19 era: A public health dilemma in Zimbabwe. Int. J. Infect. Dis..

[19] Haider N., Osman A.Y., Gadzekpo A., Akipede G.O., Asogun D., Ansumana R., Lessells R.J., Khan P., Hamid M.M.A., Yeboah-Manu D. (2020). Lockdown measures in response to COVID-19 in nine sub-Saharan African countries. BMJ Glob. Health.

[20] Greenwood B. (2014). The contribution of vaccination to global health: Past, present and future. Philos. Trans. R. Soc. B Biol. Sci..

[21] Peretti-Watel P., Larson H.J., Ward J.K., Schulz W.S., Verger P. (2015). Vaccine hesitancy: Clarifying a theoretical framework for an ambiguous notion. PLoS Curr..

[22] UN News COVID-19 Vaccination “Wildly Uneven and Unfair”: UN Secretary-General. https://news.un.org/en/story/2021/02/1084962.

[23] Duke Global Health Institute (2021). Ensuring Everyone in the World Gets a COVID Vaccine. https://globalhealth.duke.edu/news/ensuring-everyone-world-gets-covid-vaccine.

[24] Peter Mwai (2021). BBC News. Covid-19: Which Countries in Africa Are Administering Vaccines?. https://www.bbc.com/news/56100076.

[25] Gavi The Vaccine Alliance COVAX Explained. https://www.gavi.org/vaccineswork/covax-explained.

[26] WHO News COVAX Announces New Agreement, Plans for First Deliveries. https://www.who.int/news/item/22-01-2021-covax-announces-new-agreement-plans-for-first-deliveries.

[27] Relief Web: COVAX Expects to Start Sending Millions of COVID-19 Vaccines to Africa in February. https://reliefweb.int/report/world/covax-expects-start-sending-millions-covid-19-vaccines-africa-february.

[28] Heywood M. South Africa Faces Serious Setback in its AstraZeneca Vaccination Campaign—Government Turns to Plan B. https://www.dailymaverick.co.za/article/2021-02-07-south-africa-faces-serious-setback-in-its-astrazeneca-vaccination-campaign-government-turns-to-plan-b/.

[29] Daily Maverick South Africa to Give First COVID-19 Vaccine Doses to President, Health Workers. https://www.dailymaverick.co.za/article/2021-02-17-south-africa-to-give-first-covid-19-vaccine-doses-to-president-health-workers/.

[30] Columbus Mavhunga (2021). VoA News. Zimbabwe Rolls Out Coronavirus Vaccination Program. https://www.voanews.com/covid-19-pandemic/zimbabwe-rolls-out-coronavirus-vaccination-program.

[31] MoHCC (2021). Zimbabwe COVID-19 SitRep. http://www.mohcc.gov.zw/index.php?option=com_phocadownload&view=category&id=13&Itemid=744.

[32] Mukandavire Z., Nyabadza F., Malunguza N.J., Cuadros D.F., Shiri T., Musuka G. (2020). Quantifying early COVID-19 outbreak transmission in South Africa and exploring vaccine efficacy scenarios. PLoS ONE.

[33] GAVI How South Africa Is Preparing for its COVID-19 Vaccine Introduction. https://www.gavi.org/vaccineswork/how-south-africa-preparing-its-covid-19-vaccine-introduction.

[34] IOL 52% of South Africans Don’t Want Covid Vaccine, Despite SA Securing Initial Batch. https://www.iol.co.za/personal-finance/insurance/52-of-south-africans-dont-want-covid-vaccine-despite-sa-securing-initial-batch-f45373f0-6199-46ab-8207-fb632706b8e5.

[35] Tozivepi S.N., Mundagowa P., Tirivavi M., Maponga B., Mugwagwa N., Magande P., Mutseyekwa F., Makurumidze R. (2020). Covid-19 Vaccine Hesistancy Survey Preliminary Report.

[36] UNICEF, Collaborating Centre for Operational Research and Evaluation (CCORE) (2011). Apostolic Religion, Health and Utilization of Maternal and Child Health Services in Zimbabwe. https://www.unicef.org/zimbabwe/reports/apostolic-religion-health-and-utilization-maternal-and-child-health-services-zimbabwe.

[37] Mapingure M., Mukandavire Z., Chingombe I., Cuadros D., Mutenherwa F., Mugurungi O., Musuka G. (2021). Understanding HIV and associated risk factors among religious groups in Zimbabwe. BMC Public Health.

[38] Salama P., McIsaac M., Campbell J. (2019). Health workers’ strikes: A plea for multisectoral action. Bull. World Health Organ..

[39] Dodzo M.K., Mhloyi M. (2017). Home is best: Why women in rural Zimbabwe deliver in the community. PLoS ONE.

[40] Prophet Mboro Speaks against Covid 19 Vaccine. https://www.youtube.com/watch?v=q8aV52XcAdc.

[41] Taylor S., Khan M., Muhammad A., Akpala O., van Strien M., Morry C., Feek W., Ogden E. (2017). Understanding vaccine hesitancy in polio eradication in northern Nigeria. Vaccine.

[42] BBC News (2021). Covid: South Africa Variant Now “Dominant” in Zimbabwe. https://www.bbc.com/news/world-africa-56084685.

[43] Islam A.N., Laato S., Talukder S., Sutinen E. (2020). Misinformation sharing and social media fatigue during COVID-19: An affordance and cognitive load perspective. Technol. Forecast. Soc. Chang..

[44] Ward J.K., Peretti-Watel P., Verger P. (2016). Vaccine criticism on the Internet: Propositions for future research. Hum. Vaccines Immunother..

[45] WHO (2020). Managing the COVID-19 Infodemic: Promoting Healthy Behaviours and Mitigating the Harm from Misinformation and Disinformation. https://www.who.int/news/item/23-09-2020-managing-the-covid-19-infodemic-promoting-healthy-behaviours-and-mitigating-the-harm-from-misinformation-and-disinformation.

[46] SABC News (2021). Healthcare Workers Express Mixed Reaction over Halting of COVID-19 Vaccine. https://www.sabcnews.com/sabcnews/healthcare-workers-express-disappointment-over-halting-of-covid-19-vaccine/.

[47] Opera News Nurses “Reluctant” to Take Vaccine. https://www.operanewsapp.com/zw/en/share/detail?news_id=95c123bdff4bacb4356fbe2512f8558c&news_entry_id=54c22dcd210227en_zw&open_type=transcoded&from=newslite&request_id=share_request.

[48] News18 “Meant to Corrupt DNA”: South Africa Chief Justice Under Fire for Remarks About COVID Vaccines Being from Devil. https://www.news18.com/news/world/meant-to-corrupt-dna-south-africa-chief-justice-under-fire-for-remarks-about-covid-vaccines-being-from-devil-3170279.html.

[49] Grossman S., Phillips J., Rosenzweig L.R. (2018). Opportunistic accountability: State-society bargaining over shared interests. Comp. Political Stud..

[50] Renne E.P. (2014). Parallel dilemmas: Polio transmission and political violence in northern Nigeria. Afr. J. Int. Afr. Inst..

[51] Eghtessadi R., Mukandavire Z., Mutenherwa F., Cuadros D., Musuka G. (2020). Safeguarding gains in the sexual and reproductive health and AIDS response amidst COVID-19: The role of African civil society. Int. J. Infect. Dis..

[52] Sallam M. (2021). COVID-19 Vaccine Hesitancy Worldwide: A Concise Systematic Review of Vaccine Acceptance Rates. Vaccines.

[53] Schwarzinger M., Watson V., Arwidson P., Alla F., Luchini S. COVID-19 vaccine hesitancy in a representative working-age population in France: A survey experiment based on vaccine characteristics. Lancet Public Health.

[54] Caserotti M., Girardi P., Rubaltelli E., Tasso A., Lotto L., Gavaruzzi T. (2021). Associations of COVID-19 risk perception with vaccine hesitancy over time for Italian residents. Soc. Sci. Med..

[55] Murphy J., Vallières F., Bentall R.P., Shevlin M., McBride O., Hartman T.K., McKay R., Bennett K., Mason L., Gibson-Miller J. (2021). Psychological characteristics associated with COVID-19 vaccine hesitancy and resistance in Ireland and the United Kingdom. Nat. Commun..

[56] MoHCC Comprehensive National HIV Communications Strategy for Zimbabwe: 2019–2025. https://www.prepwatch.org/resource/national-comms-strategy-zim/.

[57] Kilanowski J.F. (2017). Breadth of the socio-ecological model. J Agromed..

[58] Desmon S. Stigma Related to COVID-19 May Thwart Prevention Efforts. https://ccp.jhu.edu/2021/02/08/covid-stigma-prevention-ivory-coast/.

[59] USAID, PSI (2020). “COVID is Closer than you Think. Take Care!” Campaign Kicks Off. https://zw.usembassy.gov/wp-content/uploads/sites/178/Raramo-ineTariro-nePEPFAR_-The-PEPFAR-Zimbabwe-E-Newsletter-September-002.pdf.

[60] ParkerWhite (2019). Importance of Brand Positioning in the Age of Healthcare Consumerism. https://www.parkerwhite.com/insights/brand-positioning-healthcare-consumerism/.

[61] MoHCC (2015). Creating Demad for VMMC. A New Model for Understanding. https://drive.google.com/file/d/146FhKw7V8v0aE1U92WEbNWyoZ1ySxNbT/view.

[62] Anuradha Gupta Opinion: Why We Need the Conscience Keepers to End This Pandemic. https://www.devex.com/news/opinion-why-we-need-the-conscience-keepers-to-end-this-pandemic-97388.

[63] Puri N., Coomes E.A., Haghbayan H., Gunaratne K. (2020). Social media and vaccine hesitancy: New updates for the era of COVID-19 and globalized infectious diseases. Hum. Vaccines Immunother..

[64] Rzymski P., Borkowski L., Drąg M., Flisiak R., Jemielity J., Krajewski J., Mastalerz-Migas A., Matyja A., Pyrć K., Simon K. The Strategies to Support the COVID-19 Vaccination with Evidence-Based Communication and Tackling Misinformation. https://www.comminit.com/global/content/strategies-support-covid-19-vaccination-evidence-based-communication-and-tackling-misinf.

[65] Catalan-Matamoros D., Peñafiel-Saiz C. (2019). How is communication of vaccines in traditional media: A systematic review. Perspect. Public Health.

[66] BBC News Coronavirus (2020). Pop star MP Bobi Wine sings Covid-19 Alert for the World. https://www.bbc.com/news/av/world-africa-52057381.

[67] ICAP News (2020). ICAP Invites Zimbabwean Superstars to Record COVID-19 Anthem. https://icap.columbia.edu/news-events/icap-invites-zimbabwean-superstars-to-record-covid-19-anthem/.

[68] Lahijani A.Y., King A.R., Gullatte M.M., Hennink M., Bednarczyk R.A. (2021). HPV Vaccine Promotion: The church as an agent of change. Soc. Sci. Med..

[69] Afolabi A.A., Ilesanmi O.S. (2021). Dealing with vaccine hesitancy in Africa: The prospective COVID-19 vaccine context. Pan Afr. Med. J..

